# An optimized low-pressure tourniquet murine hind limb ischemia reperfusion model: Inducing acute ischemia reperfusion injury in C57BL/6 wild type mice

**DOI:** 10.1371/journal.pone.0210961

**Published:** 2019-01-24

**Authors:** Marius Drysch, Christoph Wallner, Sonja Verena Schmidt, Felix Reinkemeier, Johannes Maximilian Wagner, Marcus Lehnhardt, Björn Behr

**Affiliations:** Department of Plastic Surgery, BG University Hospital Bergmannsheil, Ruhr University Bochum, Bürkle-de-la-Camp Platz 1, Bochum, Germany; Medical University of Graz, AUSTRIA

## Abstract

Acute ischemia reperfusion injury in skeletal muscle remains an important issue in several fields of regenerative medicine. Thus, a valid model is essential to gain deeper insights into pathophysiological relations and evaluate possible treatment options. While the vascular anatomy of mice regularly prevents sufficient vessel occlusion by invasive methods, there is a multitude of existing models to induce ischemia reperfusion injury without surgical procedures. Since there is no consensus on which model to prefer, this study aims to develop and evaluate a novel, optimized low-pressure tourniquet model. C57BL/6 mice underwent an ischemic procedure by either tourniquet or invasive artery clamping. A sham group served as control. With exception of the sham group, mice underwent 2 hours of ischemia followed by 4 hours of reperfusion. Groups were compared using microcirculatory and spectroscopic measurements, distinctions in tissue edema, histological and immunohistochemical analyses. Both procedures led to a significant decrease in tissue blood flow (- 97% vs. - 86%) and oxygenation (- 87% vs. - 75%) with a superiority of the low-pressure tourniquet. Tissue edema in the tourniquet cohort was significantly increased (+ 59%), while the increase in the clamping cohort was non-significant (+ 7%). Haematoxylin Eosin staining showed significantly more impaired muscle fibers in the tourniquet group (+ 77 p.p. vs. + 11 p.p.) and increased neutrophil infiltration/ROI (+ 51 vs. + 8). Immunofluorescence demonstrated an equal increase of p38 in both groups (7-fold vs. 8-fold), while the increase in apoptotic markers (Caspase-3, 3-Nitrotyrosine, 4-Hydroxynonenal) was significantly higher in the tourniquet group. The low-pressure tourniquet has been proven to produce reproducible and thus reliable ischemia reperfusion injury. In addition, significantly less force was needed than previously stated. It is therefore an important instrument for studying the pathophysiology of ischemia reperfusion injury and for the development of prophylactic as well as therapeutic interventions.

## Introduction

Ischemia reperfusion (I/R) injury in skeletal muscle is a well-known problem in many medical disciplines. Besides the importance in fields like trauma surgery and vascular injury, it plays an important role in free autologous tissue transfer [1,2]. Apart from the local component, I/R injury can also lead to systemic inflammatory response, multiple organ dysfunction syndrome and even death [2,3].

In order to study underlying mechanisms and gain deeper insights into pathophysiological relations, a valid I/R model is essential. Moreover, it is needed to allow evaluation of possible treatment options. Currently, there are several studies available that investigate new therapeutic targets and methods for preventing I/R injury based on animal experiments [4]. The fact that different studies use different methods to induce I/R injury inevitably impairs the reproducibility of those findings. Hence, it is crucial to have access to a standardized model as basis for future investigations.

There are several approaches to simulate an I/R situation *in vivo*. One of the main models to conduct research in composite tissue I/R is the hind limb mouse model. Many authors present open surgical methods to interfere with the arterial perfusion of the murine leg [5–10]. One major drawback occurring in the murine clamping model is the vascular anatomy of the mouse with a variety of vascular collaterals. The only sufficient invasive hind limb clamping model seems to be achieved through arterial ligation of the common iliac artery proximal of the cranial gluteal artery ramification [11]. While the biceps femoris muscle receives branches from the cranial and caudal gluteal artery and the quadriceps femoris muscle from the iliacofemoral artery, the adductor muscles receive blood supply from the internal iliac artery [11]. Side branches from these muscles act as collaterals and feed the distal caudal femoral artery as well as the saphenous artery, which both arise from the femoral artery. Still, methods for inducing acute and subacute murine hind limb ischemia are described with ligation of the main vessel branch distally of the inguinal ligament [5,12–14].

Regarding the non-invasive murine models of I/R injury, the range of techniques varies from inflatable tourniquets to cord-based models [15–17]. Moreover, there are different authors utilizing a McGivney Hemorrhoidal Ligator (MHL) or an Orthodontic Rubber Band (ORB) to induce ischemia [18,19]. While MHLs have shown decreasing tension over time, ORBs are able to establish a steady tension for 90 minutes [19]. Besides the fact that in some cases ischemia time frames over 90 minutes are required, ORBs can only be adjusted in steps [19] and therefore prevent exact adaption to altered conditions like varying hind limb circumferences. Bonheur et al [15] used a digital strain gauge to maintain the same pressure levels. Interestingly, the used pressures appear disproportionate considering that a mouse leg barely weighs 1 g while a tension of 200 g was applied to occlude blood flow. In this scenario, this tension equates a force of approximately 2 N. The approach of Sönmez et al [20] will be discussed later. In general, the used amounts of pressure differ greatly among the different tourniquet models [15–17]. In order to minimize collateral tissue damage and animal suffering, it is crucial to use only the least pressure necessary to induce I/R injury.

Given the addressed points and the current lack of available studies that directly compare an invasive to a noninvasive method, we sought to develop a reliable non-invasive model of I/R injury. For this purpose, we built a smoothly adjustable cord-based tourniquet and compared it to an existing invasive model. The overall goal of this work was therefore to present a standardized model for research in I/R injury in the murine hind limb with minimal animal harm.

## Material and methods

### Animal experiments

All animal experiments were approved by the IACUC LANUV NRW (The Ministry for Environment, Agriculture, Conservation and Consumer Protection of the State of North Rhine-Westphalia), permit number: 84–02.04.2016.A045. C57BL/6J mice were obtained from Charles River and kept with unlimited access to water and standard laboratory chow at a 12 h light/dark cycle. Littermates of both sexes at the median age of 12 (± 1) weeks were used for all experiments. All surgical procedures were performed under inhalation anesthesia with isoflurane (3% for induction, 0.8% - 1.8% for maintenance) and buprenorphine (0.05 mg/kg s.c.). To maintain a body temperature of 37°C, mice were restrained on a heating pad for the whole duration of the intervention.

### Open clamping and closed tourniquet method

**Fig 1:**
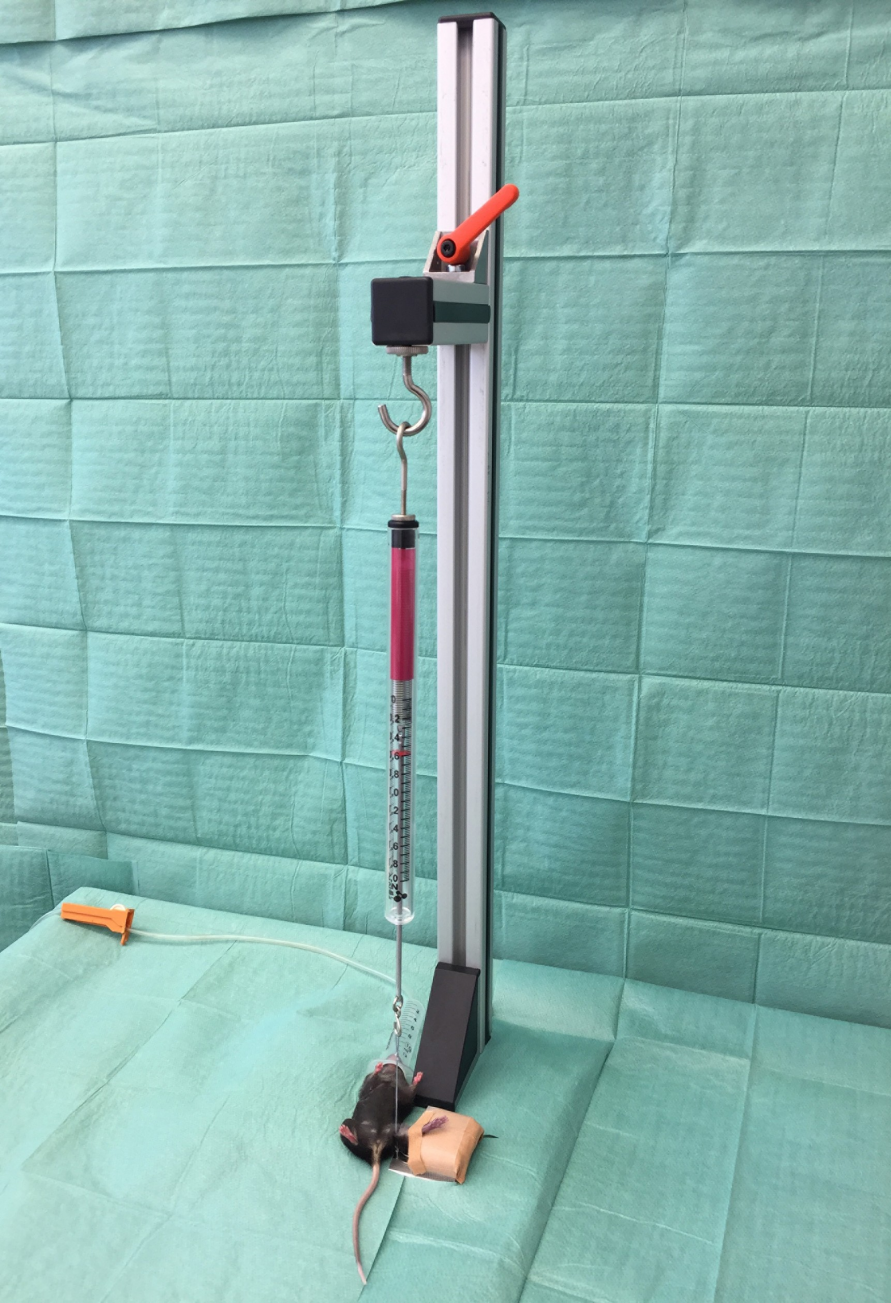
Low-pressure tourniquet.

40 Mice (24–27 g) were randomized in two experimental (n = 16) and one sham group (n = 8). After reaching the appropriate depth of anesthesia hind limbs were shaved and disinfected. Subsequently, one of the following models was used. For the open clamping model, an incision was performed on the proximal leg near to the inguinal region. Next, the femoral artery and vein were exposed and occluded right behind the inguinal ligament by using an arterial clamp. After 2 hours the clamp was removed, and the skin was closed with 5–0 Prolene interrupted sutures. Mice could recover from anesthesia during the reperfusion time of 4 hours. Afterwards euthanasia was performed by cervical dislocation and the leg was harvested. The low-pressure tourniquet (Fig 1) was structured as follows: A cord was attached to an aluminum plate on the one side and to a newton meter on the other side while the newton meter was attached to a hook. The hook could be moved freely on a splint in order to precisely reproduce the necessary amount of force. To induce ischemia, the cord was wrapped around the hind limb and, except for the microcirculatory and spectroscopic experiments, a force of 0.6 N (kg∙m∙s^-2^) was used to cause occlusion of the vessels. After 2 hours the tourniquet was removed to start reperfusion. After 4 hours of reperfusion mice were euthanized by cervical dislocation and the tissue was harvested. Control group mice were anesthetized for 2 hours without any interventions and euthanized 4 hours later. For microcirculatory and spectroscopic measurements no control mice were needed.

### Tissue edema

In order to investigate the amount of edema, a wet to dry ratio was measured. Immediately after euthanasia the leg was cut off *in toto*, weighed and placed in an oven at 55°C for 48 hours. Subsequently, the leg was weighed again and wet to dry ratio was calculated.

### Microcirculatory measurements

Microcirculatory measurements were performed using the O2C device (LEA Medizintechnik, Gießen, Germany) which combines laser Doppler and tissue spectroscopy. This device measures blood flow (in arbitrary units), tissue oxygen saturation (in percent) and relative hemoglobin content (in arbitrary units) continuously. By using LFX-36 probes (LEA Medizintechnik) these parameters were assessed at a depth of 2 mm. After shaving the leg the sensor was attached. For both methods, values were recorded 10 minutes before occlusion of the vessels. In case of the tourniquet method, starting force was set to 0.2 N increasing every 5 minutes by 0.2 N up to a total force of 2 N. Thereafter the force was released completely, and reperfusion was measured. In the clamping group mice were treated equally, except for the fact that the exerted force was not increased. All measurements were performed by the same investigator and under standardized conditions (temperature-controlled room at 22°C without direct light exposure).

### Spectroscopic measurements

For acquisition of tissue oxygenation and near-infrared (NIR) perfusion, the hyperspectral camera system TIVITA Tissue (Diaspective Vision GmbH, Pepelow, Germany) was utilized. The system covers a 500–1000 nm spectral range, and it was used as described by the manufacturer with a working distance of 50 cm. The force was administered as described in the microcirculatory measurements. All measurements were performed by the same investigator and under standardized conditions (temperature-controlled room at 22°C without direct light exposure).

### Tissue preparation and histological procedures

The harvested gastrocnemius muscle was fixed in 4% paraformaldehyde for 20–24 hours, then hydrated, embedded in paraffin and cut into 6–8 μm serial sections. Tissue samples were stained with haematoxylin and eosin for analysis of tissue damage and neutrophil infiltration. For immunohistochemical stainings of Caspase-3 (rabbit, polyclonal IgG; SantaCruz Biotechnology, sc-7148, 1:100, Caspase-3(H-277)), 3-Nitrotyrosine (mouse, monoclonal IgG_2a_, SantaCruz Biotechnology, sc-32757, 1:200, Nitrotyrosine (39B6)), Phospho-p38 MAPK (rabbit, monoclonal, IgG, Cell signaling, #4511S, 1:500, Phospho-p38 MAPK (Thr180/Tyr182) (D3F9) XP) and 4-Hydroxynonenal (rabbit, polyclonal, abcam, ab46545, 1:200, Anti-4 Hydroxynonenal antibody) slides were incubated at 70°C for 60 min. Then slides were deparaffinized, rehydrated and subsequently incubated with 0.125% Proteinase K at 37°C for 15 minutes. After a short washing step in PBS, sections were treated with blocking serum for 30 minutes, washed again in PBS and afterwards incubated with primary antibody diluted in blocking solution overnight at 4°C. After washing with PBS, a rabbit or goat secondary antibody conjugated with AlexaFluor594 or mouse IgG kappa binding protein (SantaCruz Biotechnology, sc-516179, 1:2000, m-IgGκ BP-CFL 647) was used for detection. Afterwards the sections were stained with 4′,6-diamidino-2-phenylindole (DAPI) and subsequently mounted with Fluoromount Aqueous Mounting Medium (Sigma Aldrich). Images for immunofluorescence were taken with a fluorescence microscope (Olympus IX3-Series). In order to analyze stainings, four regions of interest per section were chosen (1,000×1,000 Px). By using the Adobe Magic Wand Tool (settings: tolerance 60%; noncontiguous) immunohistochemical positive stained pixels were selected automatically and divided by countable nuclei. Afterwards a mean value was calculated.

For measuring the infiltration of neutrophils, four regions of interest per section (stained with H/E) were chosen (1,000×1,000 Px), and neutrophils were counted by three independent observers. For the morphometric analysis, an unbiased sampling procedure was applied. The fraction of normal muscle cells was calculated by measuring the cross-sectional diameters of fibers in four regions of interest in transverse sections (stained with H/E) as reported before [21]. Cells with a diameter within 10% of the control group values were considered as “normal cells”. Percentage of normal cells was calculated accordingly.

### Statistical analysis

Results of the study are presented as mean ± standard error of the mean (SEM) of at least three independent experiments. P-values were calculated by student’s t-test comparing two groups and ANOVA if comparing more than two groups after evaluation of a normal distribution through the Kolmogorov-Smirnov goodness of fit test. Statistical significances were set at a p-value < 0.05.

## Results

### Low-pressure tourniquet leads to a stronger decrease in hind limb perfusion than femoral artery clamping

**Fig 2:**
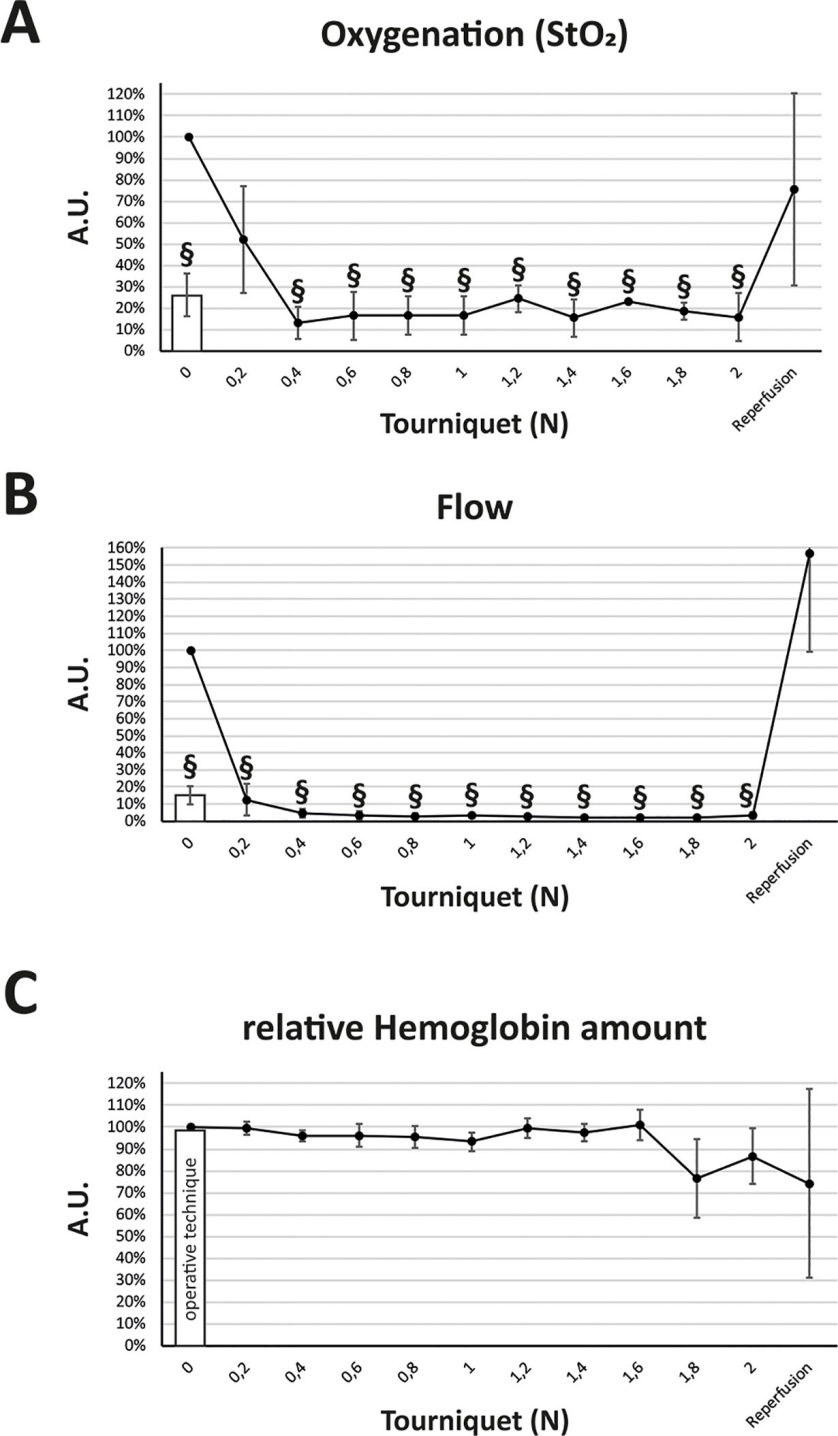
Microcirculatory measurement of oxygenation, flow and relative hemoglobin amount in the tourniquet and operative clamping model. (A) Using the O2C device, relative oxygenation of the murine hind limb was significantly reduced at a force of 0.4 N with no further reduction by increasing pressure. The operative clamping model (bar) showed a significant reduction but not as high as the low-pressure tourniquet. (B) Microvascular flow was significantly decreased at a force of 0.2 N and upwards compared to the control as well as in the operative technique (bar). (C) The relative hemoglobin amount was not significantly changed by either the operative clamping technique or increasing tourniquet pressure. Results are shown as means ± SEM. P-value: # < 0.05; § < 0.001; (two sample t-test), n = 4.

First, we systematically investigated the influence of different amounts of pressure on hind limb perfusion with the tourniquet method. As shown in Fig 2, already a force of 0.2 N almost immediately decreased perfusion to 12% (± 9%, p < 0.01), while a force of 0.4 N reduced it to 5% (± 2%, p < 0.001). At a force of 0.6 N perfusion reached 3.6% (± 2%, p < 0.001). During the ischemic period using the open method, the lowest perfusion measured was 10% (mean: 14% ± 4%, p < 0.001) which equates three times the mean flow observed using the closed method at a force of 0.6 N. The post-ischemic blood flow was increased in mice which underwent the closed ischemia induction. Five minutes after tourniquet release it was 157% (± 58%, p > 0.05). Oxygenation in the tourniquet cohort reached a minimum of 13% (± 7.5%, p < 0.001) at a force of 0.4 N. Mean oxygenation level in the open clamping group was 27% (± 11%, p < 0.001) after vessel occlusion. There were no significant changes regarding the relative hemoglobin amount.

### Spectroscopic assessments validate microcirculatory measurements

**Fig 3:**
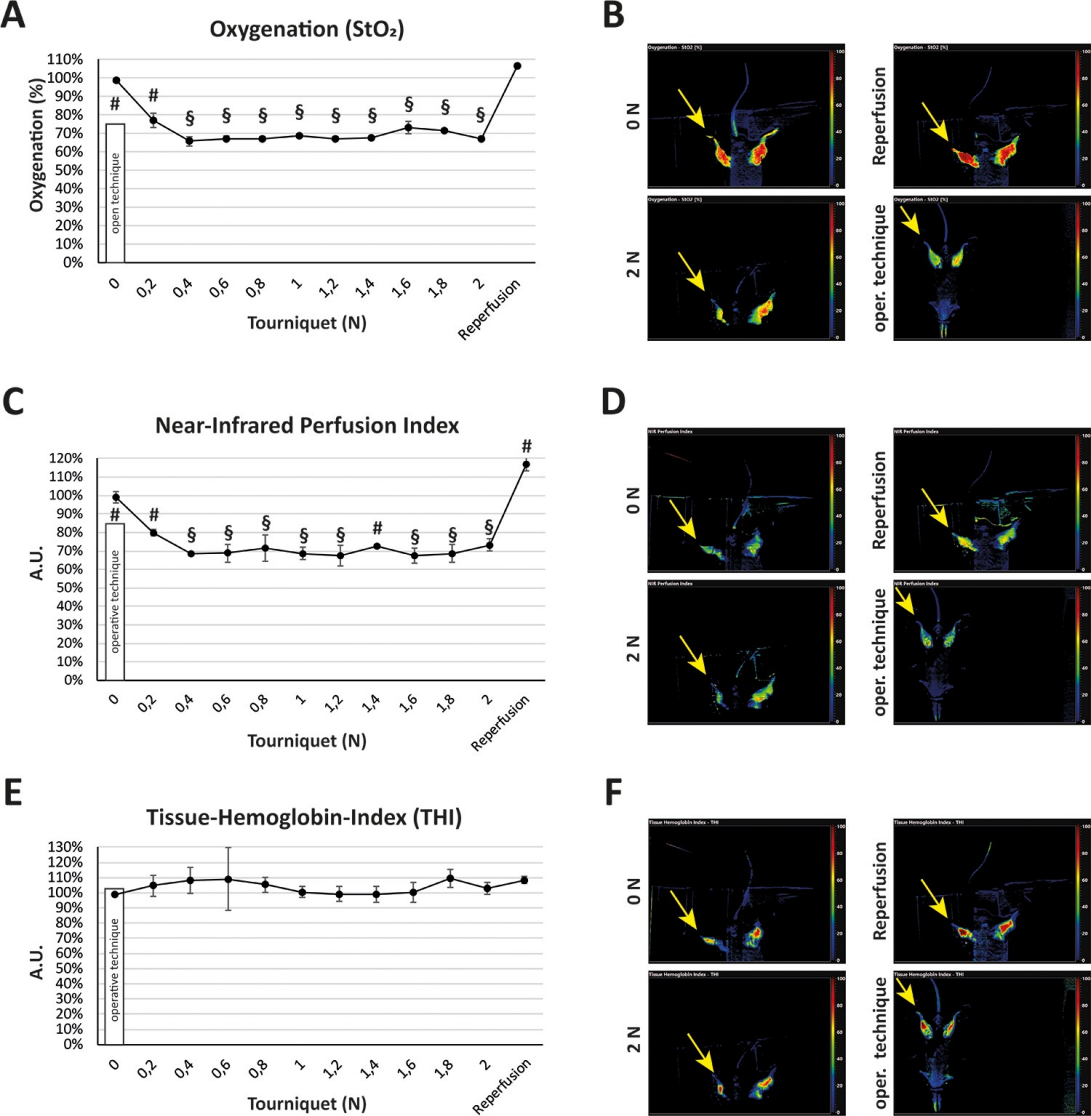
Measurement of oxygenation, perfusion and tissue hemoglobin in the tourniquet and operative clamping model with a hyperspectral camera system. (A) Using the TIVITA Tissue system, acquisition of the tissue Oxygenation (StO2) showed a significant reduction of oxygenation in the low-pressure tourniquet group at a force of 0.4 N with no further reduction of oxygenation by rising pressure. The low-pressure tourniquet group showed a higher reduction of oxygenation than the operative clamping method. (B) Live images of the oxygenation visualization exemplary at a force of 0 N, 2 N, after reperfusion and with the open clamping technique. The yellow arrow marks the compromised hind limb. (C) Measurement of the near-infrared (NIR) perfusion index showed a significant steady reduction of the hind limb perfusion starting with a force of 0.4 N. In the tourniquet group this force showed a significantly higher reduction of perfusion than the operative clamping technique. (D) Live images of the NIR exemplary at a force of 0 N, 2 N, after reperfusion and with the open clamping technique. The yellow arrow marks the compromised hind limb. (E) Measurement of Tissue-Hemoglobin-Index showed no significant change either in the tourniquet group or in the operative clamping group compared to control (0 N). (F) Live images of the Tissue-Hemoglobin-Index exemplary at a force of 0 N, 2 N, after reperfusion and with the open clamping technique. The yellow arrow marks the compromised hind limb. Results are shown as means ± SEM. P-value: # < 0.05; § < 0.001 (two sample t-test), n = 4.

In order to confirm the microcirculatory measurements, we used a hyperspectral camera system (Fig 3). Oxygenation dropped to a minimum of 66% (± 3%, p < 0.001) at a force of 0.4 N in the low-pressure tourniquet group, while open clamping led to an oxygenation of 77% (± 3%, p < 0.05). At the same force (0.4 N), the near-infrared perfusion index showed a minimum of 68% (± 1%, p < 0.001), whereas the clamping method reduced perfusion to 84% (± 3%, p < 0.05). The Tissue-Hemoglobin-Index was not affected in any of both groups. Perfusion increased to 117% (± 4%, p < 0.05) after releasing the tourniquet.

### Open clamping does not induce edema formation, while the low-pressure tourniquet causes a 1.5-fold increase in tissue fluid

**Fig 4:**
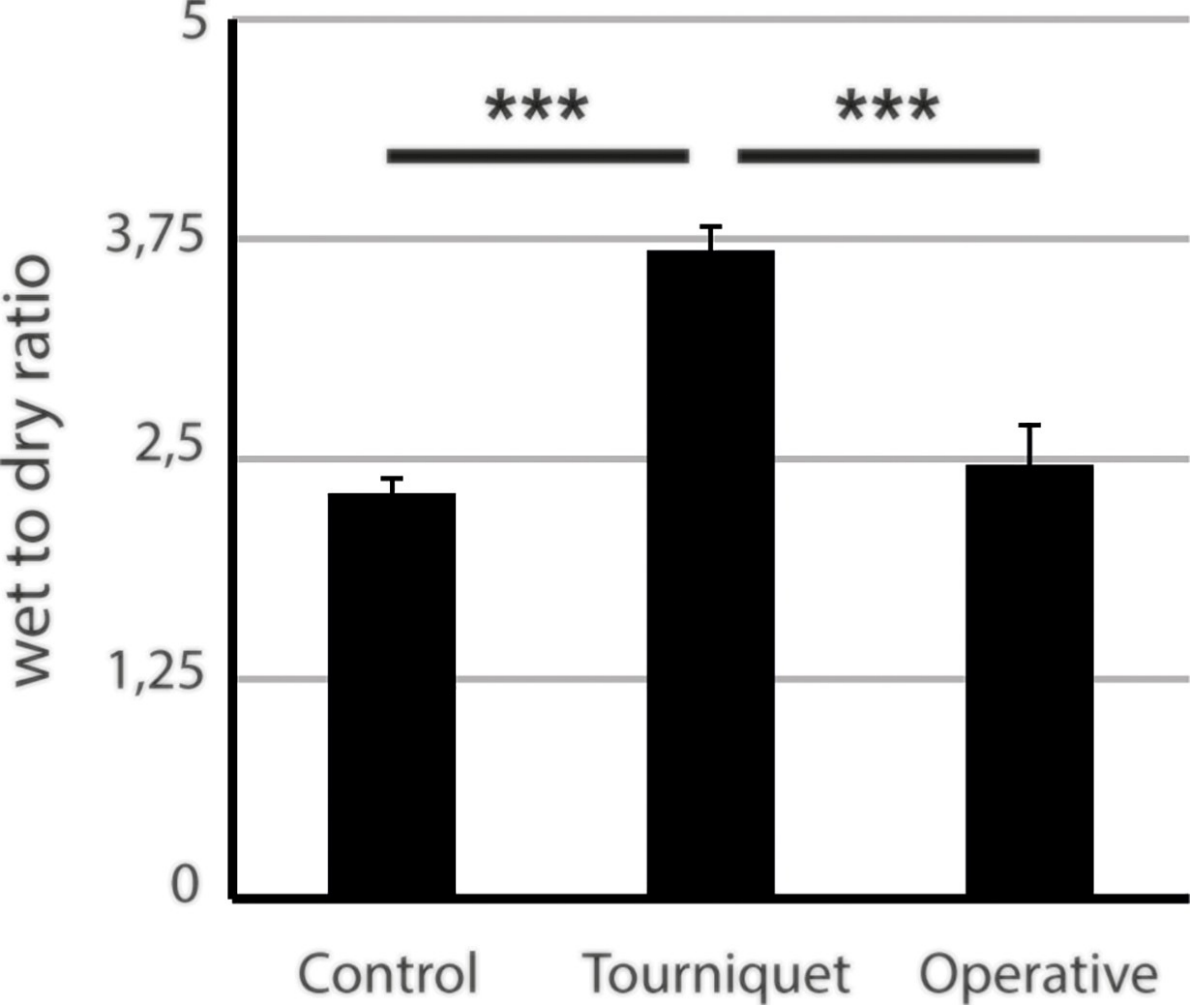
Measurement of wet to dry ratio in the low-pressure tourniquet group and operative clamping cohort. After 2 hours of ischemia and 4 hours of reperfusion the low-pressure tourniquet group showed a significant increase in edema compared to both sham and operative clamping. Results are shown as means ± SEM. P-value: *** < 0.001; (ANOVA), n = 4.

We used a modified wet to dry ratio measurement to quantify edema size (Fig 4). While there was only a non-significant difference in the operative clamping group compared to the control group (+ 7%, p > 0.05), edema in the low-pressure tourniquet group was significantly increased (+ 59%, p < 0.001).

### Low-pressure tourniquet triggers inflammatory response and leads to destruction of muscle fibers

**Fig 5:**
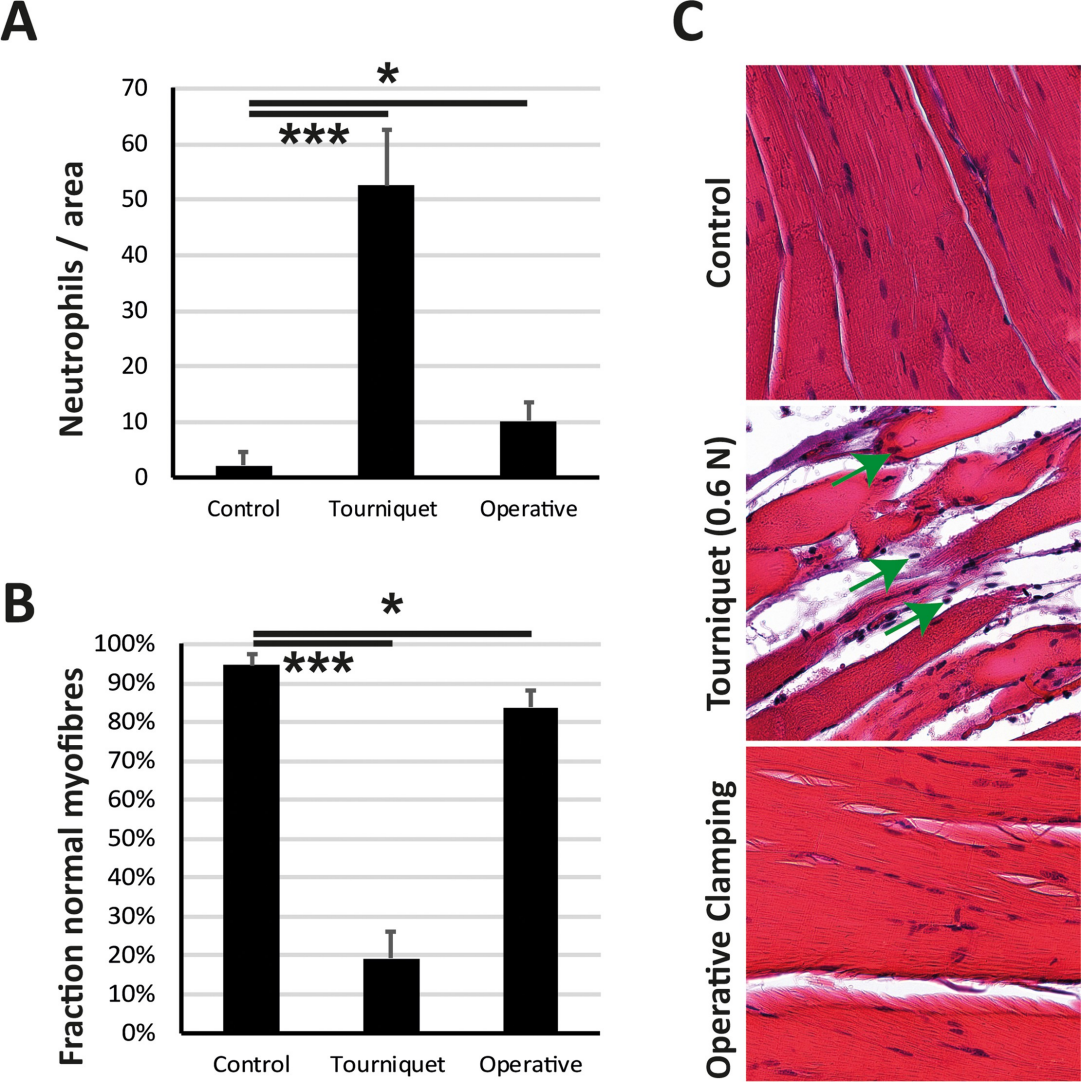
Low-pressure tourniquet leads to destruction of muscle fibers and neutrophil infiltration. After ischemia of 2 hours and reperfusion of 4 hours **(A)** Neutrophils per 2000×2000 Pixels were measured. The low-pressure tourniquet led to a significant increase in neutrophil infiltration compared to control (sham) animals. The operative clamping of the femoral artery did not show the same significant increase in neutrophil infiltration. **(B)** Fraction of normal myofibers was significantly lower in the tourniquet cohort compared to control and operative clamping. **(C)** Histological images at a magnification of 100× showed a higher neutrophil infiltration (neutrophils are marked with a green arrow) in the tourniquet group compared to control and operative clamping. Results are shown as means ± SEM. P-value: * < 0.05; *** < 0.001; (ANOVA), n = 4.

The degree of tissue damage was assessed using a haematoxylin eosin staining (Fig 5). Evaluation was based on microscopic appearance of muscle fibers as well as neutrophil infiltration. The fraction of normal muscle fibers was reduced in the operative clamping group (- 11 p.p. ± 5 p.p., p < 0.05) compared to the sham group but not as much as in the tourniquet group (- 76 p.p. ± 7 p.p., p < 0.001). Compatible with this, the increase in neutrophil infiltration was by far greater in the tourniquet group (+ 51 ± 10, p < 0.001) than in the operative clamping group (+ 8 ± 3, p < 0.05).

### Low-pressure tourniquet initiates the expression of apoptotic markers

**Fig 6:**
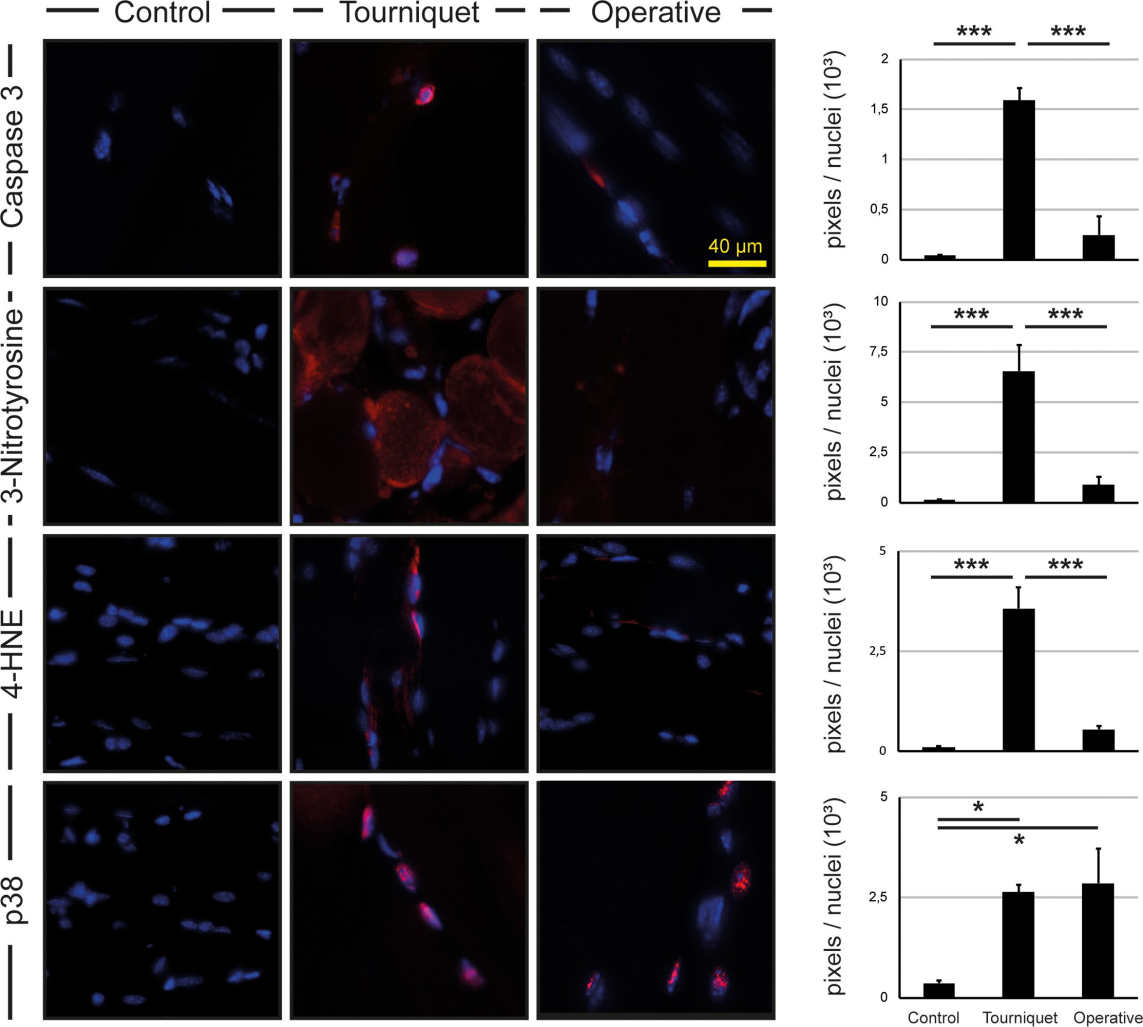
Immunofluorescence of gastrocnemius muscle in tourniquet cohort and operative clamping group. Stainings of Caspase-3, 3-Nitrotyrosine and 4-Hydroxynonenal showed an enhanced signal (red) in mice which underwent the low-pressure tourniquet method compared to control and operative clamping. Levels of p38 were elevated in both the tourniquet and the operative clamping group. Illustrations show regions of 1000×1000 pixels each. Results are shown as means ± SEM. Scale bar: 40 μm. P-value: * < 0.05; *** < 0.001; (ANOVA), n = 4.

Next we sought to investigate markers of apoptosis and oxidative stress. Therefore, we performed immunohistochemical stainings for Caspase-3, 3-Nitrotyrosine, 4-Hydroxynonenal (4-HNE) and Phospho-p38 MAPK (p38) (Fig 6). Stainings for Caspase-3 (40-fold and 7-fold, p < 0.001), 3-Nitrotyrosine (56-fold and 8-fold, p < 0.001) and 4-HNE (40-fold and 7-fold, p < 0.001) showed increased signals in the low-pressure tourniquet group compared to sham and operative clamping. Levels of p38 were increased in the low-pressure tourniquet and open clamping group compared to control (8-fold and 7-fold, p < 0.05).

## Discussion

The aim of this study was to develop a low-pressure tourniquet that induces reproducible and therefore reliable I/R injury while minimizing collateral damages and thereby animal harm. As a major finding, considerably less pressure than previously stated was needed to decrease tissue blood flow in the tourniquet group. Since we used a cord with a contact surface of 25 mm × 1 mm on the mouse leg, it calculates to 2.4 N∙cm^-2^. Bonheur et al report the use of 0.2 kg which approximately equates a force of 2 N [15] and, given the same contact surface, a pressure of 8 N∙cm^-2^. Further studies using an inflatable tourniquet report the use of up to approximately 290 mmHg (3.9 N∙cm^-2^) [16,17]. It can consequently be assumed that direct pressure damage is reduced significantly.

The microcirculatory findings showed a minimal tissue blood flow at a force of 0.4 N and are further supported by the spectroscopic measurements in which oxygenation levels dropped to a minimum at the same force. Furthermore, both techniques indicated an advantage of the tourniquet. The increased post-ischemic blood flow is consistent with other studies [22]. Since we occluded the artery as well as the vein in either experimental group, both models could have been susceptible to hemorrhagic infarction which then could also have implicated disturbances in measuring edema formation. For the clamping group this can easily be negated, since there was no significant edema formation. This is further supported by the absence of alterations in the relative hemoglobin amount and Tissue-Hemoglobin-Index. In the tourniquet group the same observations were made regarding these parameters. However, in this case we conversely observed edema formation. In accordance with the histological and immunohistochemical assessments, this is most likely a result of I/R injury. Still, the conditions including the time period used for the spectroscopic and microcirculatory experiments differed slightly from the conditions used for the other experiments. Thus, the possibility remains that there was a certain amount of unspecific edema formation that was not related to the actual I/R injury. While in other publications the wet to dry ratio may have increased more due to reperfusion injuries, it is important to note that in these publications only the pure muscle has been weighed. In order not to distort any results while preparing and processing the muscle, we weighed the whole leg. Since tissues like skin and bones are not expected to show a relevant weight loss through the processing, this inevitably leads to a lower ratio.

Even though tissue blood flow was significantly decreased in the clamping group, only very few macroscopic indicators of I/R injury were exhibited, and edema formation was non-significant. Instead, our immunohistochemical findings underline the increased apoptotic activity in the tourniquet group and go hand in hand with results of other publications on I/R injury. While it is commonly known that Caspase-3 is a crucial marker for apoptotic cell death [23,24], 3-Nitrotyrosine has been proven to be a marker of reactive oxygen species and therefore oxidative stress [25–27]. The high levels of p38 in both the clamping and the tourniquet group suggest that in either cohort regulatory response mechanisms are activated upon ischemic conditions. Considering the macroscopic and immunohistochemical findings, elevated levels of p38 in the tourniquet group are explained by the fact that p38 is known to lead to I/R injury through the p38MAPK/MK2 signaling pathway [28,29]. However, increased levels of p38 activity in the clamping group are highly improbable a result of severe I/R injury, since there was no further evidence to support this assumption. An activation of p38 is also described in chronic ischemic conditions [30] and during Remote Ischemic Preconditioning (RIPC) [31–33] which is an experimental approach in I/R injury prevention. Briefly, a cycle of repeated short I/R situations is used to increase blood flow which then results in a higher ischemic tolerance even in other regions of the body. One could speculate that the open clamping method leads to subacute ischemic conditions and therefore causes RIPC-like conditions. 4-Hydroxynonenal is a product of lipid peroxidation and is also known to play a role in cellular redox stress and apoptotic cell death through activation of the caspase cascade [34,35]. Therefore, its elevation is compatible with elevated Caspase-3 levels and underlines the inflicted I/R injury.

A point to consider is the influence of varying hind limb circumferences. Sönmez et al [20] continuously used a Laser-Doppler Flowmetry assisted model to ensure sufficient induction of ischemia. This may be a reasonable approach as we used microcirculatory and spectroscopic measurements ourselves to validate the tourniquet. However, in order to develop an I/R injury model for common practice, one should take into consideration that such equipment is expensive and therefore not readily available for every research group. The microcirculatory and spectroscopic measurements indicate a sufficient depletion of blood flow through administration of 0.4 N of force. In order to take care of potential variations in hind limb circumferences and ensure vessel occlusion, further experiments were performed using an applied force of 0.6 N. Indeed, if available, a device like a Doppler imaging system may be an option for notably altered conditions like significant changes in hind limb circumference. Translation to different mouse strains or other rodents such as rats may also belong to the potential scope. Based on the presented findings we, however, conclude that its blanket use is dispensable. Briefly summarized, our method provides a reliable model for studying hind limb I/R without cost-intensive apparatus to monitor its effects with concurrent benefits for animal welfare.

This study is limited by the fact that the selected invasive method naturally cannot be representative of all existing invasive models. Besides the used method there are further models. A different approach, for instance, is to occlude the femoral artery a second time distal to the first occlusion, additionally ligate the saphenous artery, transect the proximal femoral artery or ligate collateral branches [5,6,9,10,12]. But even if this led to a stronger reduction in blood flow, it would seem barely feasible to occlude all collateral branches. As mentioned in the introduction, the murine hind limb vascular system has numerous collaterals and interindividual disparities [11]. Complex interventions also prevent a simultaneous reopening of all vessels, as it would be necessary for investigating I/R injury. In addition, the invasiveness of identifying even the smallest vessels paired with interindividual distinctions of mice results in a poor harm-benefit ratio for the animals [36]. Considering the mentioned points, we therefore focused on a straight-forward, reliable and reproducible surgical method.

With the now introduced model, clinical problems related to ischemic conditions may be investigated. The necessity for investigation in this field is further elucidated by its applicability for a broad patient population. A vivid instance for this is total knee arthroplasty which is often carried out using a tourniquet in order to minimize blood loss and improve overview of the surgical field. However, this has been demonstrated to trigger oxidative stress and I/R injury [37,38]. Indeed, prevention and treatment of I/R injury following total knee arthroplasty has been the subject of different clinical trials [39,40]. Overall, there are several clinical implications of I/R injury that urge the need for further investigation. For instance, the decrease in required pressure for inducing I/R injury as shown in our study might implicate a possible effect of human RIPC with significantly lower tourniquet pressures. Down the road, findings generated with this model could eventually end up in clinical trials and help develop and validate new therapeutic approaches.

## Conclusion

The present findings confirm that the presented low-pressure tourniquet provides a reliable and reproducible method to induce acute I/R injury in the murine hind limb.

## Supporting information

S1 Dataset Supporting information containing the dataset used for the figures in the manuscript. (PDF)
