# Supplementary material for: An optimized low-pressure tourniquet murine hind limb ischemia reperfusion model: Inducing acute ischemia reperfusion injury in C57BL/6 wild type mice
Source: PLoS One. 2019 Jan 24;14(1):e0210961. doi: 10.1371/journal.pone.0210961 (PMC6345480; doi:10.1371/journal.pone.0210961)

		geschlossen	geschlossen	geschlossen	geschlossen			Mean	Stab	p
Ctrl		1	1	1	1		0	1	0	
	0,2	0,261744966	0,05380334	0,152014652	0,02688172		0,2	0,123611	0,092355	0,002
	0,4	0,07852349	0,05380334	0,037509158	0,016429211		0,4	0,046566	0,022715	0
	0,6	0,077852349	0,01546073	0,031428571	0,019130824		0,6	0,035968	0,024894	0
	0,8	0,056375839	0,019789734	0,021098901	0,016263441		0,8	0,028382	0,016259	0
	1	0,048322148	0,019789734	0,023663004	0,045922939		1	0,034424	0,0128	0
	1,2		0,017872604	0,034798535			1,2	0,026336	0,008463	0
	1,4		0,012368584	0,030842491			1,4	0,021606	0,009237	0
	1,6		0,013048856	0,031941392			1,6	0,022495	0,009446	0,001
	1,8		0,013296228	0,023516484			1,8	0,018406	0,00511	0
	2	0,059731544	0,012473717	0,034798535			2	0,035668	0,019303	0
Reperfusion offen		1,597986577	1,615213358	2,35018315	0,714605735	Reperfusion		1,569497	0,579485	
Kontrolle		0,98	1	0,99				0,99	0,008165	

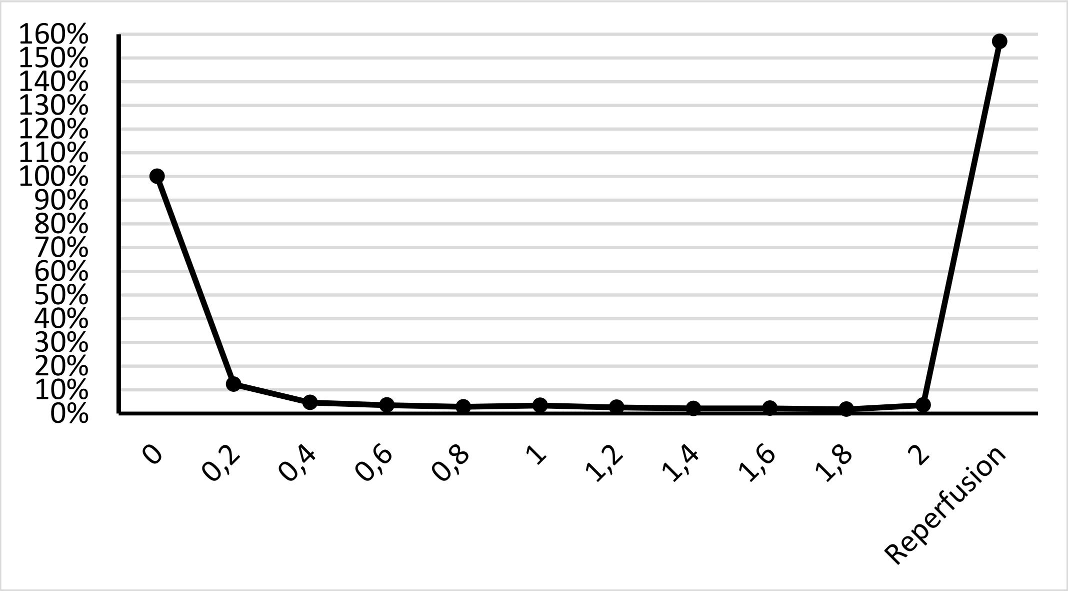

		geschlossen	geschlossen	geschlossen	geschlossen			Mean	Stab	p
Ctrl		1	1	1	1		0	1	0	
	0,2	0,680851064	0,100854701	0,561827411	0,73266743		0,2	0,51905	0,249264	0,002
	0,4	0,258156028	0,112820513	0,063045685	0,097110949		0,4	0,132783	0,074587	0
	0,6	0,062695035	0,247863248	0,043451777	0,301737592		0,6	0,163937	0,112693	0
	0,8	0,124822695	0,278632479	0,044771574	0,222488121		0,8	0,167679	0,089802	0
	1	0,10212766	0,288888889	0,06284264	0,207574654		1	0,165358	0,088811	0
	1,2		0,305128205	0,181827411			1,2	0,243478	0,06165	0
	1,4		0,241025641	0,067309645			1,4	0,154168	0,086858	0
	1,6		0,222222222	0,23715736			1,6	0,22969	0,007468	0,001
	1,8		0,227350427	0,147208122			1,8	0,187279	0,040071	0
	2	0	0,21965812	0,2538411			2	0,157833	0,112474	0
Reperfusion		0,859574468	1,188034188	0	0,973884091	Reperfusion		0,755373	0,451771	
offen		0,123887588	0,283010432	0,390977444				0,265958	0,109704	0,001
Kontrolle		0,98	1	0,99				0,99	0,008165	

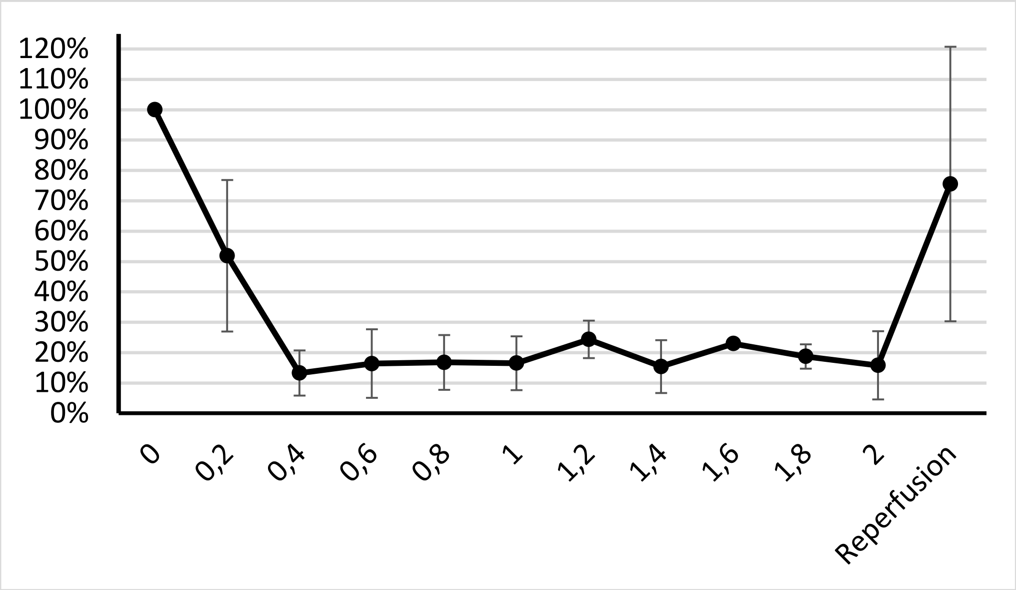

		geschlossen	geschlossen	geschlossen	geschlossen		Mean	Stab	p
Ctrl		1	1	1	1	0	1	0	
	0,2	0,985032741	0,952636282	1,022885572	1,019495706	0,2	0,995013	0,028599	0,002
	0,4	0,936389149	0,938337802	0,998706468	0,970854706	0,4	0,961072	0,025681	0
	0,6	0,88868101	0,934763181	0,995024876	1,024114144	0,6	0,960646	0,052581	0
	0,8	0,884939196	0,933869526	0,995024876	1,010160564	0,8	0,955999	0,049989	0
	1	0,88049579	0,948525469	0,995024876	0,906982686	1	0,932757	0,043363	0
	1,2		0,949061662	1,04079602		1,2	0,994929	0,045867	0
	1,4		0,934763181	1,014427861		1,4	0,974596	0,039832	0
	1,6		0,938337802	1,078208955		1,6	1,008273	0,069936	0,001
	1,8		0,945487042	0,585074627		1,8	0,765281	0,180206	0
	2	0,687122	0,933422699	0,9822452		2	0,867597	0,129162	0
Reperfusion		1,053320861	1,018766756	0	0,897156221	Reperfusion	0,742311	0,432481	
offen		1,080575384	0,934555454	0,970182992			0,995105	0,062163	0,001
Kontrolle		0,98	1	0,99			0,99	0,008165	

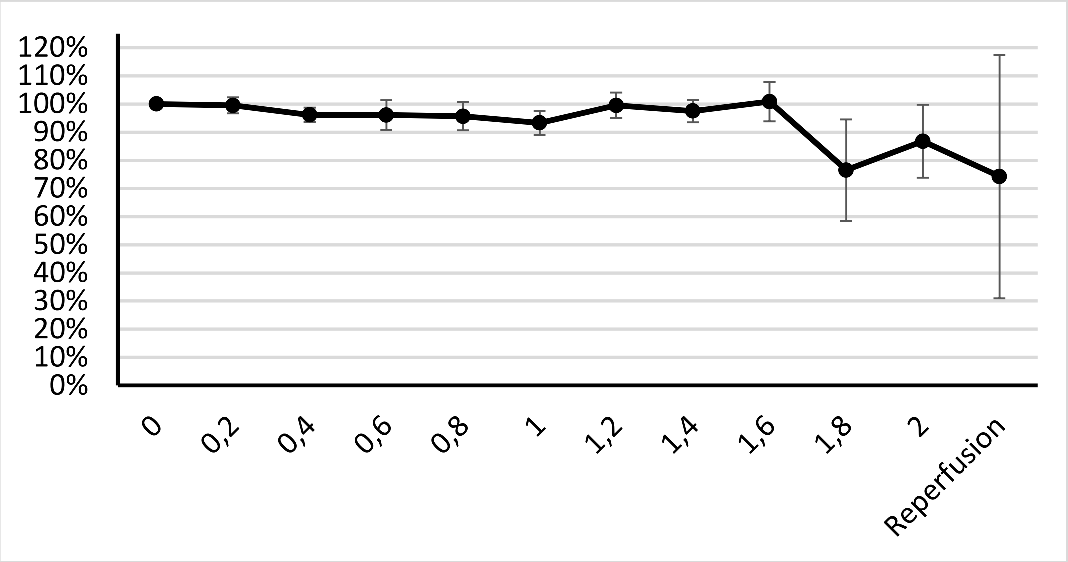

		geschlossen	geschlossen	geschlossen		Mean	Stab	p
Ctrl		1	0,95	1,02	0	0,99	0,029439	
	0,2	0,818181818	0,777777778	0,8	0,2	0,798653	0,016522	0,002
	0,4	0,682926829	0,697674419	0,673913043	0,4	0,684838	0,009794	0
	0,6	0,756097561	0,666666667	0,644444444	0,6	0,68907	0,048256	0
	0,8	0,804878049	0,625	0,717948718	0,8	0,715942	0,073449	0
	1	0,731707317	0,666666667	0,658536585	1	0,685637	0,032745	0
	1,2	0,75	0,651162791	0,622222222	1,2	0,674462	0,054705	0
	1,4	0,731707317	0,711111111	0,733333333	1,4	0,725384	0,010114	0
	1,6	0,674418605	0,625	0,725	1,6	0,674806	0,040826	0,001
	1,8	0,625	0,688888889	0,743589744	1,8	0,685826	0,048462	0
	2	0,76	0,68627451	0,75	2	0,732092	0,032654	0
Reperfusion		1,115384615	1,2	1,196078431	Reperfusion	1,170488	0,038997	
offen		0,804878049	0,875	0,829268293		0,836382	0,029066	0,001
Kontrolle		1	0,95	1,02		0,99	0,029439	

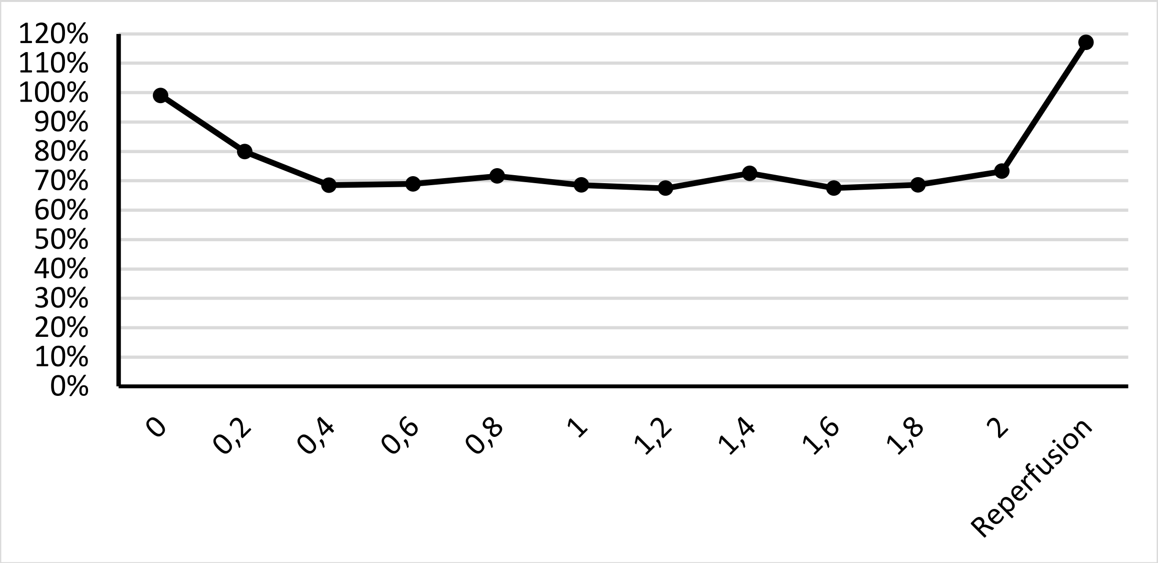

		geschlossen	geschlossen	geschlossen			Mean	Stab	p
Ctrl		0,98	1	0,99		0	0,99	0,008165	
	0,2	0,734939759	0,828571429	0,753623188		0,2	0,772378	0,04046	0,002
	0,4	0,630952381	0,693333333	0,653333333		0,4	0,659206	0,025803	0
	0,6	0,654761905	0,685714286	0,68		0,6	0,673492	0,013448	0
	0,8	0,662650602	0,6875	0,666666667		0,8	0,672272	0,010892	0
	1	0,686046512	0,695121951	0,688311688		1	0,689827	0,003857	0
	1,2	0,666666667	0,682926829	0,666666667		1,2	0,672087	0,007665	0
	1,4	0,662921348	0,681818182	0,682926829		1,4	0,675889	0,009181	0
	1,6	0,782051282	0,705128205	0,7125		1,6	0,733226	0,034655	0,001
	1,8	0,716049383	0,72	0,716049383		1,8	0,717366	0,001862	0
	2	0,680555556	0,657534247	0,682352941		2	0,673481	0,0113	0
Reperfusion		1,057471264	1,079545455	1,054347826	Reperfusion		1,063788	0,011215	
offen		0,727272727	0,78	0,8			0,769091	0,030676	0,001
Kontrolle		0,98	1	0,99			0,99	0,008165	

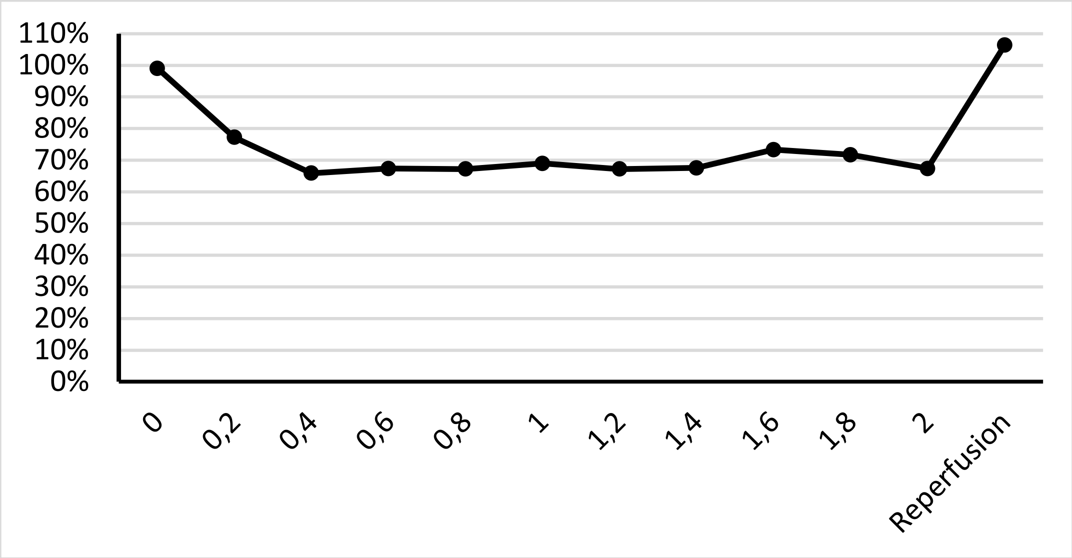

		geschlossen	geschlossen	geschlossen			Mean	Stab	p
Ctrl		0,98	1	0,99		0	0,99	0,008165	
	0,2	1,140350877	0,982758621	1,016393443		0,2	1,046501	0,067768	0,002
	0,4	0,972222222	1,180327869	1,086956522		0,4	1,079836	0,085108	0
	0,6	1,387096774	0,925925926	0,961538462		0,6	1,09152	0,209509	0
	0,8	0,985507246	1,086956522	1,093333333		0,8	1,055266	0,049395	0
	1	1,014705882	0,96	1,043478261		1	1,006061	0,034624	0
	1,2	0,942857143	0,972222222	1,061538462		1,2	0,992206	0,05047	0
	1,4	0,961538462	0,942028986	1,066666667		1,4	0,990078	0,054739	0
	1,6	1,090909091	0,935483871	0,975609756		1,6	1,000668	0,06588	0,001
	1,8	1,163265306	1,1	1,020833333		1,8	1,0947	0,058268	0
	2	1,068965517	0,972222222	1,041666667		2	1,027618	0,040725	0
Reperfusion		1,11627907	1,066666667	1,071428571	Reperfusion		1,084791	0,02235	
offen		1,144927536	0,975609756	0,962025316			1,027521	0,083204	0,001
Kontrolle		0,98	1	0,99			0,99	0,008165	

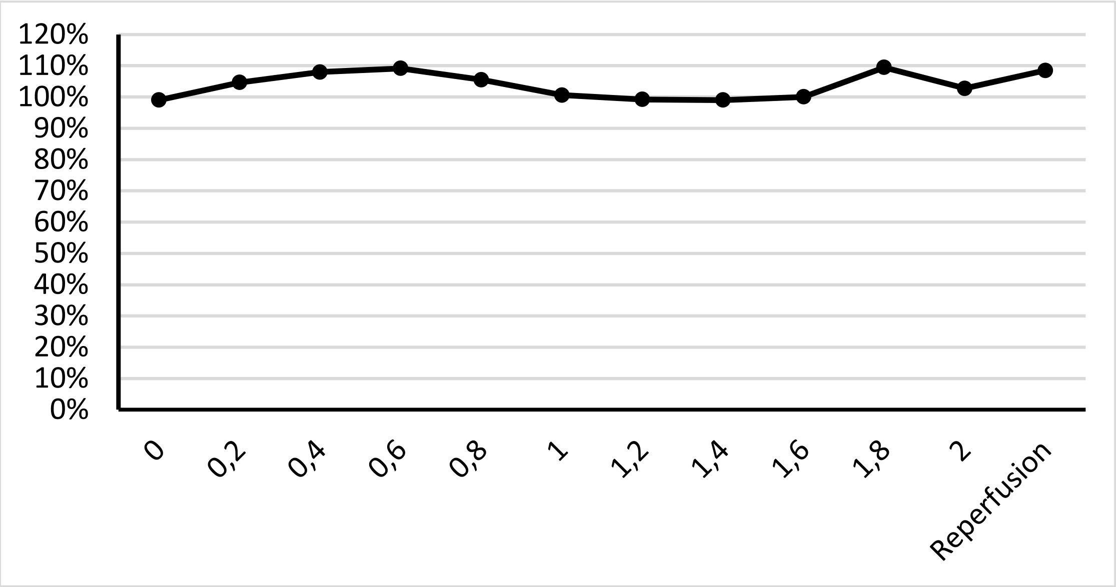

	Schale	0	Bein 0	48h	Bein 48h	Ödemgewicht
ctrl 1	13,71	14,37	0,66	14,01	0,3	0,36
ctrl 2	13,68	14,26	0,58	13,93	0,25	0,33
ctrl 3	2,025	2,95	0,925	2,41	0,385	0,54
offen 1	13,73	14,36	0,63	13,98	0,25	0,38
offen 2	13,69	14,28	0,59	13,95	0,26	0,33
offen 3	13,7	14,47	0,77	14,04	0,34	0,43
offen 4	2,06	2,96	0,9	2,38	0,32	0,58
geschlossen 1	13,71	14,76	1,05	13,98	0,27	0,78
geschlossen 2	13,68	14,59	0,91	13,94	0,26	0,65
geschlossen 3	13,7	14,54	0,84	13,93	0,23	0,61
geschlossen 4	2,036	3,38	1,344	2,4	0,364	0,98

	wet to dry
ctrl 1	2,20
ctrl 2	2,32
ctrl 3	2,40
offen 1	2,52
offen 2	2,27
offen 3	2,26
offen 4	2,81
geschlossen 1	3,89
geschlossen 2	3,50
geschlossen 3	3,65
geschlossen 4	3,69

	wet to dry MW	wet to dry SA
Control	2,31	0,08
Tourniquet	3,68	0,14
Operative	2,47	0,22

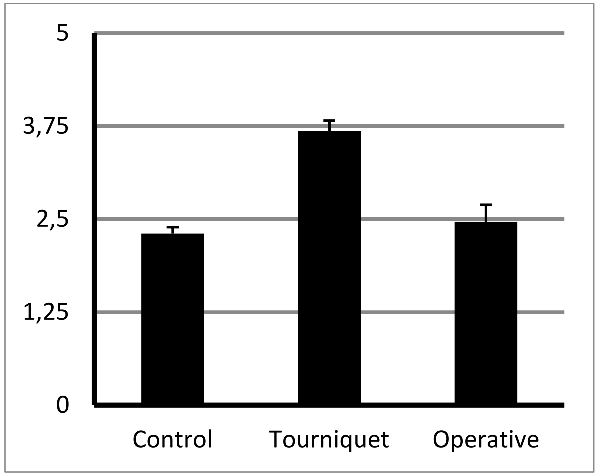

ctrl	5
ctrl	0
ctrl	4
ctrl	0
closed	45
closed	68
closed	42
closed	55
open	5
open	10
open	12
open	14

Control	2,25	2,27760839
Tourniquet	52,5	10,1612007
Operative	10,25	3,34477204

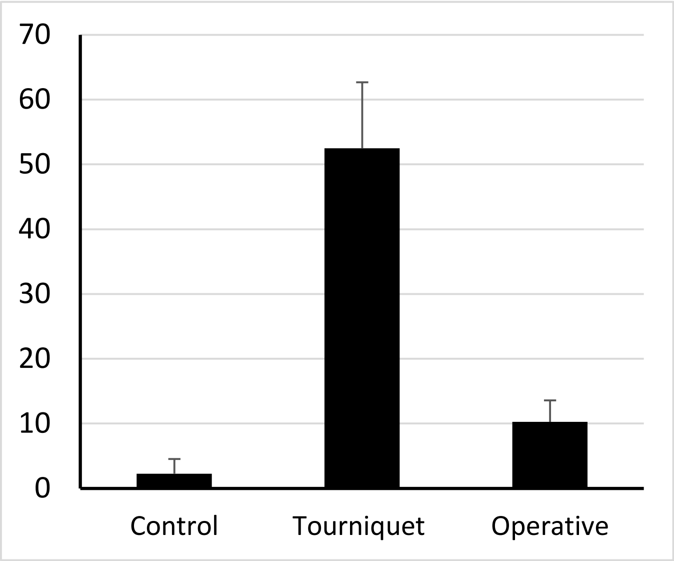

ctrl	0,9
ctrl	0,95
ctrl	0,98
ctrl	0,95
closed	0,12
closed	0,15
closed	0,3
closed	0,2
open	0,78
open	0,85
open	0,82
open	0,9

Control	0,945	0,02872281
Tourniquet	0,1925	0,06832825
Operative	0,8375	0,04380354

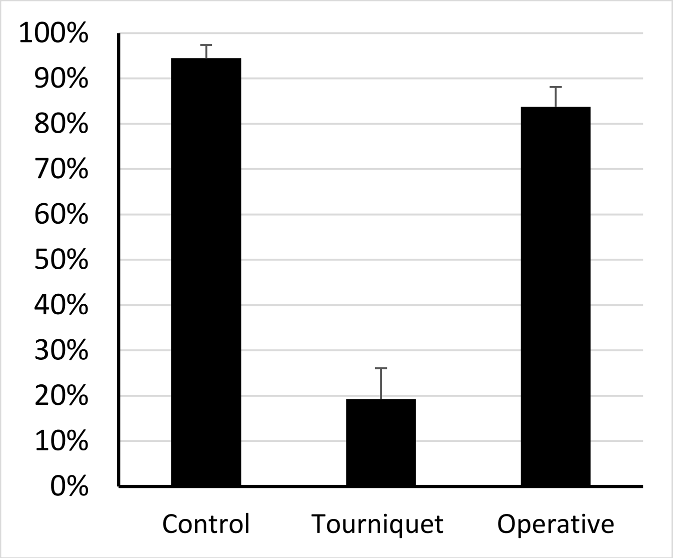

	Kern	Wert	AU			Mittelwert
Control1_1	42	27209	647,833333	Gestrichen	Ctrl	47,0435606
Control1_2	44	157	3,56818182			
Control1_3	39	0	0			
Control1_4	32	4402	137,5625			
Closed1_1	50	99435	1988,7		Closed	1414,92347
Closed1_2	59	2895	49,0677966	Gestrichen		
Closed1_3	44	58067	1319,70455			
Closed1_4	41	38391	936,365854			
Control2_1	58	0	0		Ctrl	38,8914729
Control2_2	78	14796	189,692308	Gestrichen		
Control2_3	38	0	0			
Control2_4	86	10034	116,674419			
Open1_1	33	12268	371,757576		Open	452,66699
Open1_2	27	26307	974,333333	Gestrichen		
Open1_3	41	15338	374,097561			
Open1_4	48	29383	612,145833			
Control3_1	32	0	0		Ctrl	29,8635478
Control3_2	55	10259	186,527273	Gestrichen		
Control3_3	38	2484	65,3684211			
Control3_4	45	1090	24,2222222			
Closed2_1	68	87580	1287,94118		Closed	1676,01615
Closed2_2	53	128140	2417,73585			
Closed2_3	37	14540	392,972973	Gestrichen		
Closed2_4	35	46283	1322,37143			
Control4_1	27	3568	132,148148	Gestrichen	Ctrl	40,3888889
Control4_2	28	0	0			
Control4_3	31	0	0			
Control4_4	42	5089	121,166667			
Open2_1	20	0	0		Open	0
Open2_2	27	0	0			
Open2_3	32	0	0			
Open2_4	33	0	0			

Open3_1	54	20444	378,592593		Open	283,213735
Open3_2	32	8294	259,1875			
Open3_3	24	23744	989,333333	Gestrichen		
Open3_4	36	7627	211,861111			
Closed_3_1	43	78967	1836,44186		Closed	1680,80337
Closed_3_2	45	65273	1450,51111			
Closed_3_3	33	90593	2745,24242	Gestrichen		
Closed_3_4	35	61441	1755,45714			

	Mittelwerte	Standardabweichung	
Control	39,0468675	6,12588016	
Closed	1590,581	124,224005	Closed
Open	245,293575	186,735651	

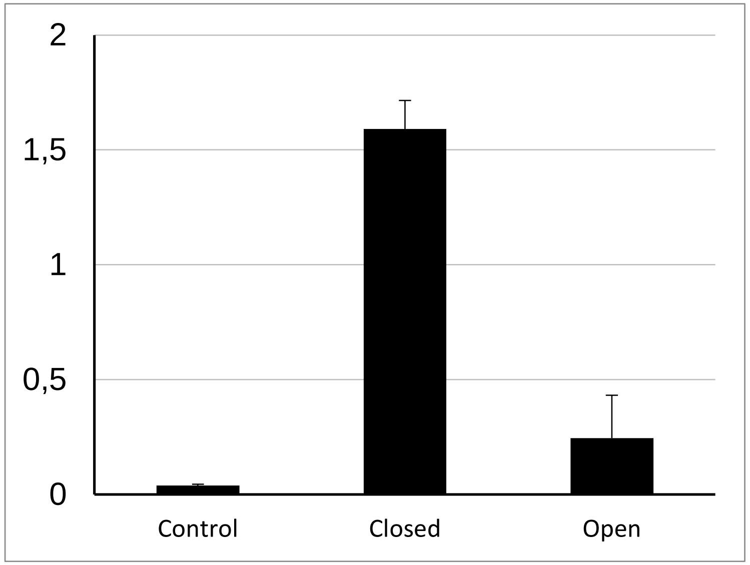

	Kern	Wert	AU		Mittelwert	
Control1_1	36	12878	357,722222		124,240741	
Control1_2	34	41224	1212,47059	Gestrichen		
Control1_3	50	0	0			
Control1_4	28	420	15			
Closed1_1	40	29251	731,275	Gestrichen	5402,48202	
Closed1_2	29	121959	4205,48276			
Closed1_3	31	243692	7861,03226			
Closed1_4	29	120087	4140,93103			
Control2_1	58	20322	350,37931		469,735111	Gestrichen
Control2_2	36	12877	357,694444			
Control2_3	40	129352	3233,8	Gestrichen		
Control2_4	38	26643	701,131579			
Open1_1	52	20517	394,557692		1174,89517	
Open1_2	55	0	0			
Open1_3	42	45099	1073,78571			
Open1_4	38	78141	2056,34211			
Control3_1	31	0	0		93,1919192	
Control3_2	37	0	0			
Control3_3	33	9226	279,575758			
Control3_4	50	221851	4437,02	Gestrichen		
Closed2_1	28	90650	3237,5	Gestrichen	5804,19196	
Closed2_2	49	222372	4538,20408			
Closed2_3	45	368259	8183,53333			
Closed2_4	24	103218	4300,75			
Closed2_5	50	255926	5118,52			
Closed2_6	42	288958	6879,95238			
Control4_1	33	812	24,6060606		130,242313	
Control4_2	42	8880	211,428571			
Control4_3	33	0	0	Gestrichen		
Control4_4	26	4022	154,692308		1120,62233	
Open2_1	42	24423	581,5			
Open2_2	32	48718	1522,4375			

Open2_3	52	39517	759,942308		
Open2_4	39	42100	1079,48718		
Open3_1	28	5786	206,642857		317,576549
Open3_2	27	62252	2305,62963	Gestrichen	
Open3_3	42	25835	615,119048		
Open3_4	31	4060	130,967742		
Closed_3_1	35	358338	10238,2286		8395,67725
Closed_3_2	52	169202	3253,88462	Gestrichen	
Closed_3_3	43	304678	7085,53488		
Closed_3_4	41	322394	7863,26829		

	Mittelwerte	Standardabweichung
Control	115,891658	16,2370629
Open	871,03135	391,978356
Closed	6534,11708	1326,49851

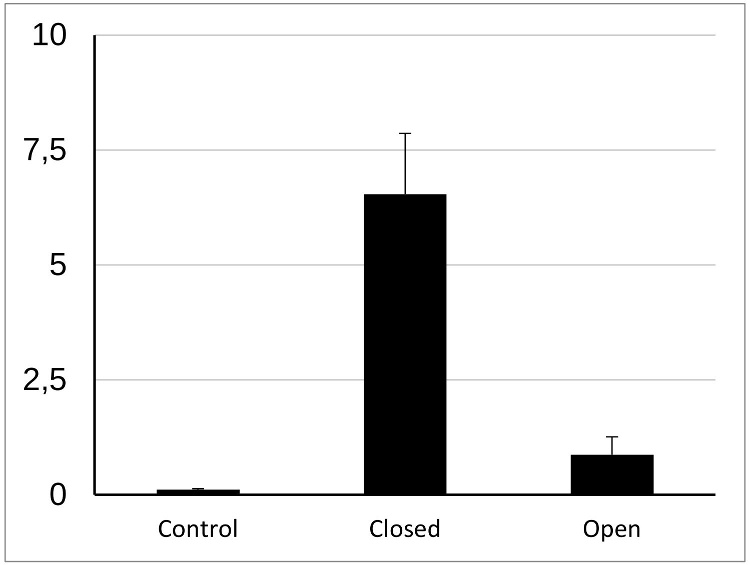

	Kern	Wert	AU		Mittelwert
Control1_1	16	0	0		101,708333
Control1_2	17	0	0		
Control1_3	33	11860	359,393939	Gestrichen	
Control1_4	16	4882	305,125		
Closed1_1	10	109016	10901,6		4175,59054
Closed1_2	18	50809	2822,72222		
Closed1_3	17	76662	4509,52941		
Closed1_4	25	129863	5194,52		
Control2_1					#DIV/0!
Control2_2					
Control2_3					
Control2_4					
Open1_1	58	36210	624,310345		542,451395
Open1_2	54	23294	431,37037		
Open1_3	49	28012	571,673469		
Open1_4	58	2047	35,2931034	Gestrichen	
Control3_1	45	2751	61,1333333		115,453914
Control3_2	55	10741	195,290909		
Control3_3	48	4317	89,9375		
Control3_4	65	27273	419,584615	Gestrichen	
Closed2_1	42	153696	3659,42857		2866,11293
Closed2_2	50	129387	2587,74		
Closed2_3	45	98837	2196,37778	Gestrichen	
Closed2_4	47	110505	2351,17021		
Control4_1	25	714	28,56		40,1935043
Control4_2	23	410	17,826087	Gestrichen	
Control4_3	30	1526	50,8666667		
Control4_4	26	1070	41,1538462		
Open2_1	35	5908	168,8	Gestrichen	633,550642
Open2_2	28	23432	836,857143		
Open2_3	25	15559	622,36		
Open2_4	23	10153	441,434783		

Open3_1	42	16778	399,47619	425,627023
Open3_2	35	13181	376,6	
Open3_3	47	35665	758,829787	
Open3_4	41	20533	500,804878	
Closed_3_1	38	150471	3959,76316	3641,88494
Closed_3_2	40	119502	2987,55	
Closed_3_3	40	145833	3645,825	
Closed_3_4	45	149403	3320,06667	

	Mittelwerte	Standardabweichung
Control	85,7852506	32,7229863
Closed	3561,19614	537,628074
Open	533,876353	85,1007495

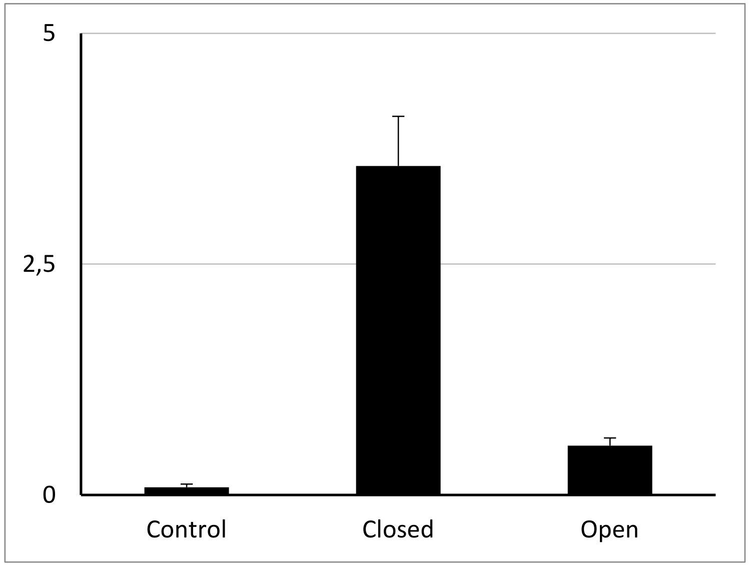

	Kern	Wert	AU		Mittelwert
Control1_1	33	0	0		429,1875
Control1_2	20	0	0		
Control1_3	29	117519	4052,37931	Gestrichen	
Control1_4	32	41202	1287,5625		
Closed1_1	25	63204	2528,16		2736,96259
Closed1_2	30	95971	3199,03333	Gestrichen	
Closed1_3	20	63034	3151,7		
Closed1_4	36	91117	2531,02778		
Control2_1					
Control2_2					
Control2_3					
Control2_4					
Open1_1	27	46670	1728,51852		2264,04189
Open1_2	32	72024	2250,75		
Open1_3	65	91631	1409,70769	Gestrichen	
Open1_4	35	98450	2812,85714		
Control3_1					
Control3_2					
Control3_3					
Control3_4					
Closed2_1	32	78667	2458,34375		2379,49248
Closed2_2	42	104101	2478,59524		
Closed2_3	39	85860	2201,53846		
Closed2_4	55	76561	1392,01818	Gestrichen	
Control4_1	28	7349	262,464286		292,808669
Control4_2	27	0	0	Gestrichen	
Control4_3	33	1944	58,9090909		
Control4_4	19	10584	557,052632		
Open2_1	35	61775	1765		2232,97691
Open2_2	33	73110	2215,45455		
Open2_3	37	60788	1642,91892	Gestrichen	
Open2_4	21	57088	2718,47619		

Open3_1	35	160002	4571,48571		4069,16377
Open3_2	42	187634	4467,47619		
Open3_3	51	161595	3168,52941		
Open3_4	49	101176	2064,81633	Gestrichen	
Closed_3_1	49	144467	2948,30612		2776,47578
Closed_3_2	37	149501	4040,56757	Gestrichen	
Closed_3_3	44	112770	2562,95455		
Closed_3_4	48	135272	2818,16667		

	Mittelwerte	Standardabweichung
Ctrl	360,998085	68,1894153
Open	2855,39419	858,358397
Closed	2630,97695	178,556529

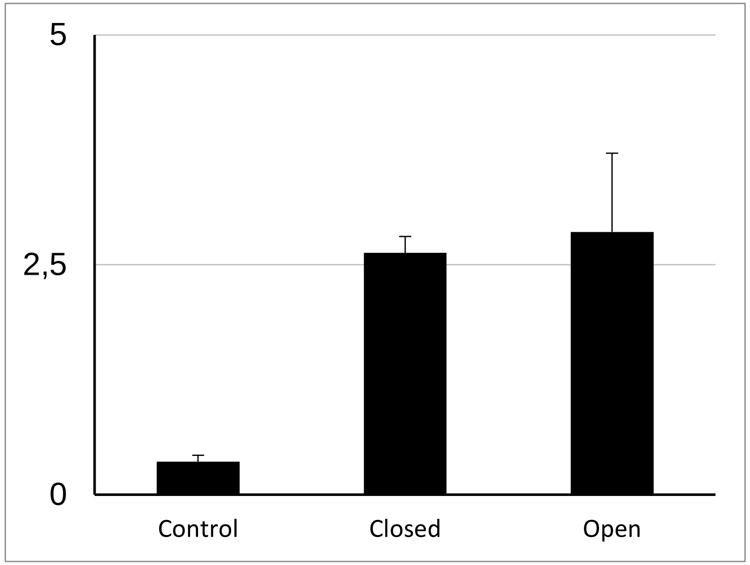

Supplement: S1 Dataset — (PDF) [file pone.0210961.s001.pdf]
